# Spatial and temporal behavioural responses of wild cattle to tropical forest degradation

**DOI:** 10.1371/journal.pone.0195444

**Published:** 2018-04-12

**Authors:** Penny C. Gardner, Benoît Goossens, Jocelyn Goon Ee Wern, Petra Kretzschmar, Torsten Bohm, Ian P. Vaughan

**Affiliations:** 1 Danau Girang Field Centre, c/o Sabah Wildlife Department, Wisma Muis, Kota Kinabalu, Sabah, Malaysia; 2 Organisms and Environment Division, Cardiff School of Biosciences, Cardiff University, Cardiff, United Kingdom; 3 Sabah Wildlife Department, Wisma Muis, Kota Kinabalu, Sabah, Malaysia; 4 Sustainable Places Research Institute, Cardiff University, Cardiff, United Kingdom; 5 Leibniz Institute for Zoo and Wildlife Research, Berlin, Germany; Gaziosmanpasa University, TURKEY

## Abstract

Identifying the consequences of tropical forest degradation is essential to mitigate its effects upon forest fauna. Large forest-dwelling mammals are often highly sensitive to environmental perturbation through processes such as fragmentation, simplification of habitat structure, and abiotic changes including increased temperatures where the canopy is cleared. Whilst previous work has focused upon species richness and rarity in logged forest, few look at spatial and temporal behavioural responses to forest degradation. Using camera traps, we explored the relationships between diel activity, behavioural expression, habitat use and ambient temperature to understand how the wild free-ranging Bornean banteng (*Bos javanicus lowi*) respond to logging and regeneration. Three secondary forests in Sabah, Malaysian Borneo were studied, varying in the time since last logging (6–23 years). A combination of generalised linear mixed models and generalised linear models were constructed using >36,000 trap-nights. Temperature had no significant effect on activity, however it varied markedly between forests, with the period of intense heat shortening as forest regeneration increased over the years. Bantengs regulated activity, with a reduction during the wet season in the most degraded forest (z = -2.6, Std. Error = 0.13, p = 0.01), and reductions during midday hours in forest with limited regeneration, however after >20 years of regrowth, activity was more consistent throughout the day. Foraging and use of open canopy areas dominated the activity budget when regeneration was limited. As regeneration advanced, this was replaced by greater investment in travelling and using a closed canopy. Forest degradation modifies the ambient temperature, and positively influences flooding and habitat availability during the wet season. Retention of a mosaic of mature forest patches within commercial forests could minimise these effects and also provide refuge, which is key to heat dissipation and the prevention of thermal stress, whilst retention of degraded forest could provide forage.

## Introduction

Tropical forests harbour much of the world’s biodiversity and provide important ecosystem services [1], yet are increasingly impacted by selective logging and conversion to agricultural plantations [2]. Much of the logging is unsustainable and results in large-scale forest degradation [1]. Pivotal changes in forest structure follow timber extraction and subsequent harvesting cycles, resulting in progressive reduction of the biomass, alongside collateral damage to the structure and composition of residual vegetation, and the disruption to the restoration dynamics of the forest [3–5]. Timber harvesting activity also results in soil compaction, damage to the seed bank, increased runoff and pollution of waterways, and increased air and water temperatures as shading is reduced [5,6].

Our knowledge of the impacts of selective logging on vertebrate biodiversity is limited to a few taxa [2], and of the studies conducted, the majority focus on biodiversity metrics such as species richness [2,7], rather than behavioural adaptations to post-logging conditions. Activity and behaviour are important components of a species’ ecological strategy and have profound implications for reproduction and survival [8], however they are seldom used to estimate the magnitude of habitat disturbance. Considering that mammals are generally more sensitive to forest disturbance than taxa such as plants, invertebrates and birds [9], the impacts of timber harvesting may be evident in their behaviour sooner, making them more useful taxa for assessing the consequences. However, quantifying the behaviour of rare species is difficult. For large vertebrates, it is likely that changes in their community are induced by the logging activity and by the subsequent decades of forest regeneration [9,10]. Most behavioural studies of the effects of selective logging upon vertebrates have concerned primates (mostly orang-utans), which show changes in energy allocation, variation in locomotion and use of the different canopy strata, and negative spatial responses to logging [11–14]. Forest disturbances are also known to affect a myriad of other mammals, reducing species occupancy [15] and population densities [16]. Conversely, increases in species abundance and richness have also been documented for some mammals [17] and birds [15].

Timber harvesting results in reduced and more patchy leaf canopy cover which increases penetration of sunlight to the forest floor, and can result in elevated ambient temperatures [18,19]. Temperature is one of the most important environmental variables governed by tree cover [19]; the ambient temperature of logged tropical forest was found to be >13°C higher than adjacent primary forest [18]. High temperatures can alter the behaviour of mammals that have a large body mass, and this can result in high heat production and a lethal rise in core body temperature [20]. In extremely high ambient temperatures, the regulation of activity is key to the dissipation of heat and maintaining fitness [21]. Dissipation of body heat can be conducted by means of wallowing and bathing [22], and seeking refuge in shaded habitat and choosing a nocturnal habit when temperatures are cooler [20,21,23]. Thermo-relief provided by a closed canopy and close proximity to water are important for large mammals [23] because they have very narrow thermal niches [21,23–25]. In contrast, post-logging structural changes can actually benefit some taxa; logging can induce the growth of lianas that may provide a valuable food source and physically link trees together, therefore facilitating arboreal access [26]. The removal of trees can increase light at the forest floor and the growth of ground vegetation favoured by large mammals [27,28], whilst the opening of logging roads creates corridors for wildlife movement [29] and increases access to foraging areas [10].

Rare species that inhabit secondary tropical forests are problematic to study directly, and non-invasive survey methods like remote camera traps may be one of the few ways to collect quantitative data on their behaviours. The endangered and little-studied Bornean banteng (*Bos javanicus lowi*) is a large wild cattle species but is difficult to detect in the forests of Sabah, Malaysian Borneo, and camera traps are the most effective method of collecting robust ecological data on this free-ranging species [30]. A highly sensitive bovid, the Bornean banteng converts to a nocturnal habit when disturbed by heavy vehicles [31] and, when logging activity commences, it retreats to undisturbed forest, sometimes pushed into higher elevations, which are left unlogged due to the unfavourable slope [32]. These closed forests are a refuge from disturbances [33].

Sabah is a global hotspot of forest degradation and, due to oil palm and timber industries, in 2009 only 8% of protected areas comprised intact forest that had not been logged since the 1970s [5]. In the absence of extensive primary tropical lowland dipterocarp forest, bantengs in Sabah are forced into secondary forests that are logged repeatedly on rotation at intervals well under the 60 years that is prescribed in most Malaysian forestry plans [5]. Consequently, the forests are scarred from harvesting activity; old logging roads and skid trails traverse all secondary forest across Sabah [5] and are still evident 20 years after logging activity has ceased (P. Gardner, pers. obs.). These roads can foster favourable conditions for plant regeneration of forage suitable for ungulates [27,28], including the growth of herbs, grasses, ferns and shrubs favoured by Bornean bantengs (Ridge, unpublished data). Bornean bantengs congregate along old logging roads [34], and are thought to utilise these areas for foraging (Ridge, unpublished data). Short-term increases in the body condition of banteng were found in individuals inhabiting forest that was recently logged using Reduced Impact Logging (RIL) [35], and RIL probably facilitated increased uptake of forage by providing short-term increased understorey growth in the first few years after logging [35]. Conversely, banteng in conventionally-logged forest had lower body condition scores for many years [35], which suggested they probably suffered the repercussions of heavy disturbance and altered forest structure decades after logging had ended.

In light of the extensive forest degradation in Sabah and induced changes in behaviour observed in tropical mammals following timber harvesting, this study focused on the impacts of logging on the Bornean banteng, and used camera traps to conduct the first behavioural study on this species. This study had aims to determine any differences in diel activity associated with different logging histories and ambient temperatures, and estimate the diurnal variation in activity budgets and habitat use. All aims were achieved using camera trap event data arising from banteng photographic captures and generalised linear mixed models and bootstrapped generalised linear models.

## Materials and methods

Research permits were granted by the Sabah Biodiversity Council, reference numbers: JKM/MBS.1000- 2/12(156) and JKM/MBS.1000-2/2 JLD.3 (18)

### Study sites

**Fig 1:**
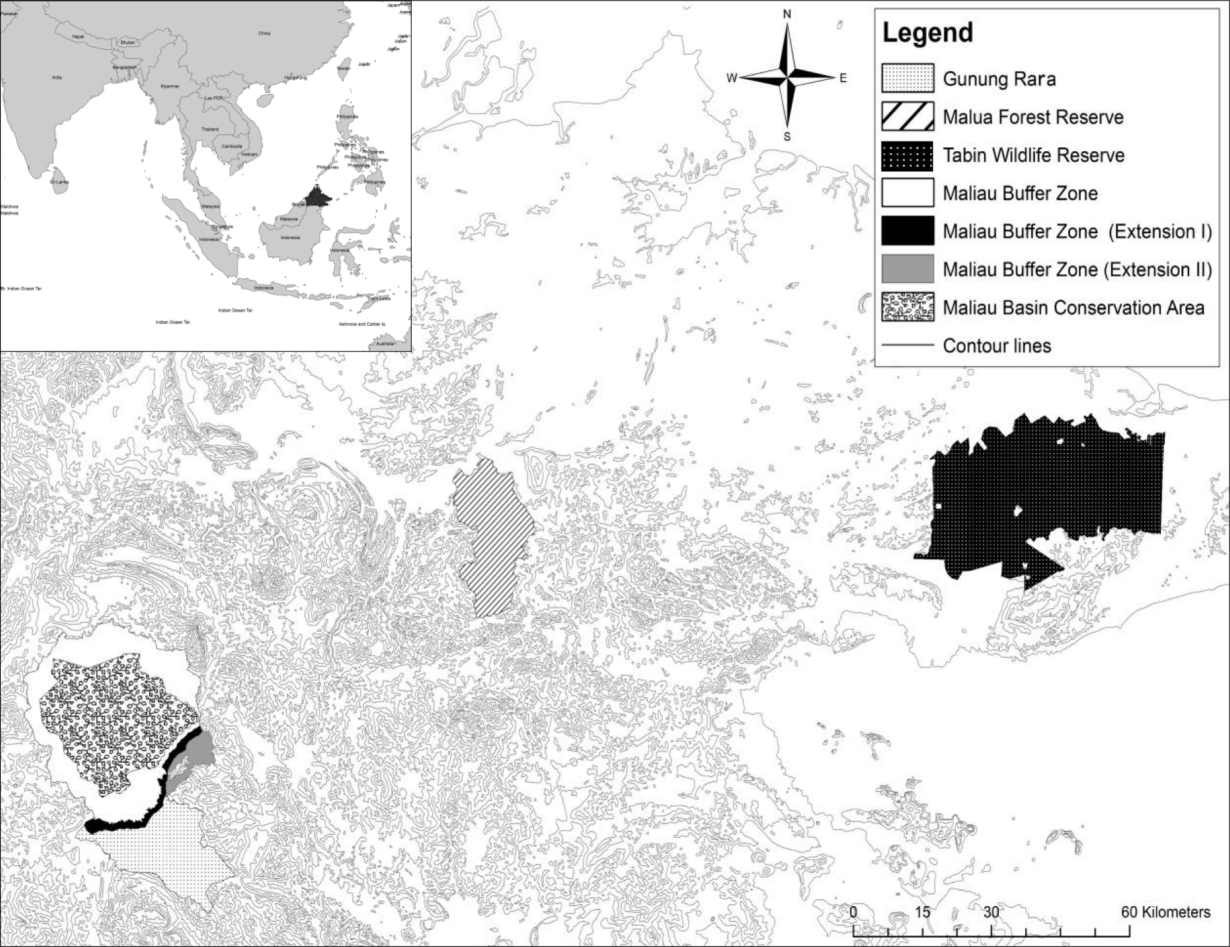
Study area. Map showing the location of Sabah (inset) on the island of Borneo in South East Asia, and the distribution of three forest reserves with different post-logging regeneration ages. East Sabah: Tabin Wildlife Reserve (TWR). Central Sabah: Malua Forest Reserve (MFR). South central Sabah: Maliau Basin Conservation Area Buffer Zone (MBCABZ) with a small portion of Gunung Rara Forest Reserve directly to the south of MBCABZ). Maps were generated using ArcGIS® software version 10.1 by ESRI, with data from Natural Earth and the Sabah Forestry Department.

This study was conducted between 2011–2013 in three secondary protected forests in the Malaysian state of Sabah (Borneo): Malua Forest Reserve (MFR; 5° N, 117° E), Maliau Basin Conservation Area Buffer Zones (MBCABZ; 4° N, 116° E) and Tabin Wildlife Reserve (TWR; 5° N, 118° E) (Fig 1). The three forests are considered to be degraded, which, in this study, refers to forests which have experienced extensive and repeated removal of timber, contain internal logging road networks with exposed and compacted substrate resulted from heavy machinery use. These forests were selected to provide a gradient of regeneration time since logging activity had ceased. MFR was in the early stages of regeneration, with no logging since 2007 (six years prior to our study). Previously, MFR underwent extensive and repetitive timber harvesting from the 1960s onwards [36] using a combination of conventional, traditional (crawler tractor), and reduced impact logging (RIL) techniques including heli-logging in higher elevations and the log-fisher method (see S1 Fig). Much of MFR is heavily degraded with a low tree density, especially for large commercial timber species. Forest gaps are smothered by climbing bamboos and vines, and hamper natural forest regeneration (New Forests Ltd, unpublished data).

MBCABZ was in a later stage of regeneration, with an estimated 17 years since logging ended in the mid-1990s [37]. It was managed as a Class II Commercial Forest Reserve until November 2015, when it was upgraded to Class I Protected Forest Reserve [38]. Our study was conducted within the south of the main buffer zone, in buffer zones extension I and II, and in a very small portion of Gunung Rara Forest Reserve. Wide old logging roads have created an open canopy under which grasses, soft vines and herbs are abundant, whilst open areas were limited and generally colonised by wild ginger, grasses, herbs, ferns, soft vines. The prevalence of mature trees burdened by vines and climbing bamboo was notably less compared to MFR and were generally confined to river banks and open areas.

TWR is protected against logging for the purpose of wildlife conservation but was extensively logged up until 1989 using conventional harvesting methods, which created a vast network of logging roads and skid trails. Twenty-three years of regeneration had elapsed since the end of logging activity and the start of our survey. Areas that were intensively logged are dominated by dense thickets of bamboo, wild ginger and rattan, whilst regeneration of lowland freshwater swamp, lowland mixed dipterocarp and seasonal freshwater swamp forest in the east has been largely unsuccessful and remains open habitat. Mature trees with large basal areas are generally confined to the deep interior of TWR, which was apparently less intensively logged due to the steeper topography and slope.

### Field methods

A total of 337 camera trap stations were deployed across the three forest reserves (Table 1) at four location types representing varying degrees of canopy cover: 1) active access roads that were used at least once every week by a vehicle, 2) old logging roads, 3) open areas such as log preparation sites, and 4) forest trails representing a closed canopy. Cameras (Reconyx HC500 and Reconyx PC800, Reconyx Inc., Wisconsin, USA) were deployed in pairs facing one another, approximately 5-10m apart and 50-150cm above the ground, to capture both sides of the animals for identification purposes. Cameras were active day and night, and were triggered by a combination of heat and motion. For every trigger, three photographs and associated ambient temperature were recorded at one-second intervals. Cameras were checked approximately every 30 days to ensure functionality and clear vegetation from the detection area, and left in-situ for a minimum period of 90 days, previously identified as super-optimal for detecting banteng [30].

**Table 1:** Summary of camera trap surveys in three regenerating logged forests in Sabah, Malaysian Borneo.

Regeneration period (approx.):		6 years	17 years	23 years
	Year logging ended	2007	1997	1989
**Authors**	**Forest name**	**Malua Forest Reserve**	**Maliau Basin Conservation Area Buffer Zones**	**Tabin Wildlife Reserve**
PCG, BG, JGEW and IV	Number of Stations	138	23	130
	Survey design	Grid & ad-hoc	Ad-hoc	Grid & ad-hoc
	Target species	Banteng	Banteng	Banteng
	Start of survey	29/03/2011	20/06/2013	29/03/2011
	End of survey	22/10/2013	17/08/2014	18/10/2012
	No. CT nights	13,304	5,134	13,951
	No. banteng events	313	158	40
	No. discounted events	20	2	0
	No. events wet season	105	79	6
	No. events dry season	188	77	34
PK and TB	Number of Stations	*N/A*	*N/A*	46
	Survey design	*N/A*	*N/A*	Ad-hoc
	Target species	*N/A*	*N/A*	Sumatran rhino
	Start of survey	*N/A*	*N/A*	18/07/2012
	End of survey	*N/A*	*N/A*	16/02/2013
	No. CT nights	*N/A*	*N/A*	4,161
	No. banteng events	*N/A*	*N/A*	8
	No. discounted events	*N/A*	*N/A*	0
	No. events wet season	*N/A*	*N/A*	8
	No. events dry season	*N/A*	*N/A*	0

Photographs were assumed to correlate with banteng activity patterns [33]. Autocorrelation was minimised in the study design and during data preparation: all stations with the exception of three were established >100m apart, whilst multiple events per station were discounted if they occurred within the same hour [39–41]. Where possible, all individuals were identified using natural markings [30], and multiple events of the same individuals during the same hour were discounted to minimise pseudo-replication, even if arising from different stations.

### Modelling of diel activity and temperature data

Data analysis comprised two parts: i) an overview of the time series activity and temperature data over 24 hours within the three forests, ii) an investigation into how the hourly climatic variation affected the expression of behaviours and variation in habitat use, and iii) differences in activity between the three periods of the day. Event data was extracted from camera trap photographs and compiled into a time series for activity, which was prepared by stratifying event data according to the forest of occurrence (MFR, MBCABZ and TWR), season (wet: January to March and October to December, and dry: April to September), period (nocturnal: 00:01–04:00 and 20:01–00:00 hours, crepuscular: 04:01–06:00 and 18:01–20:00 hours, and diurnal: 06:01–18:00 hours), two-month interval (e.g. Jun-Jul), and two-hour interval (e.g. 00:01–02:00), which were explanatory categorical variables. For this study, activity was defined as the number of independent camera trap events. To assess the ambient temperatures within banteng forest habitat associated with their activity and habitat use, we extracted temperature (°C), recorded by the camera traps, from every banteng event and assembled the data following the same method as above; the mean ambient temperature was estimated for each two-hour interval. Two-hour periods were used for aggregating the data as they provided the best compromise between temporal detail and the degree of replication. We used a combination of generalised linear models (GLM) and generalised linear mixed models (GLMM) in R 3.2.3 [42] to estimate banteng activity. Using the package lme4 [43], random effects Poisson GLMMs using the log link function were contrasted to assess the variation in banteng activity and ambient temperature; we constructed a random intercept model, with fixed effects for temperature, season and time of day and a random effect for time of day, and contrasted this with a random intercept and slope model, which had an additional inclusion of a random effect for temperature that was allowed to vary according to the time of day, as we suspected that the effect of temperature may be greater at midday hours. To assess the variation in ambient temperature between forests, we constructed two Gamma GLMMs using the identity link for a random intercept model with both a fixed and random effect for time of day, and a random intercept and slope model whereby temperature was allowed to vary with time of day, as above. Model selection was conducted using Akaike’s information criterion, and model validation using plots of the residuals. To derive robust estimates of mean two-hourly activity frequencies, associated ambient temperature, and the 95% confidence limits of each estimate, GLM were fitted once using the package MASS [44] and then bootstrapped to 400 permutations, following the protocols of [45,46]; A log link and Poisson family was used to model activity as functions of forest, season, and time of day, with an interaction between forest and time, whilst the same model structure was used to model ambient temperature but with an identity link and Gamma family.

### Modelling of activity budgets and habitat use

Activity budgets for each two-hourly interval were created following [47] and [24], using the duration of the event calculated from the date/time of the first and last photograph. Behaviours were classified according to three categories: 1) foraging, 2) travelling, and 3) resting (standing, seemingly aimless walking around or laying down) at each location: 1) active access roads, 2) old logging roads, 3) open areas, and 4) forest trails. The selection of a behaviour was based on the majority of the herd performing one behaviour, therefore only one behaviour could be performed per event. Following the same protocol for modelling the diel activity data, we fitted Poisson log link GLMMs to the above three behaviour categories and four locations to estimate the effect of temperature and time of day upon the activity budgets and habitat use in the three regenerating forests. We also fitted three Poisson log link GLMs (one to each behaviour), and four Gamma identity link GLMs (one to each location), and bootstrapped to obtain the two-hourly estimates and confidence limits for activity budgets and habitat use. We assessed if the ambient temperature of each regenerating forest, influenced the duration and the type of behaviours expressed and the location preferences by using Spearman’s p tests.

### Periodic preferences of the diel cycle

To determine the daylight hour preferences of bantengs occupying the three forests, random effects GLMMs with a log link and Poisson family were fitted to the activity frequency, with period of the day (with three factors: nocturnal, crepuscular and diurnal) as a fixed effect, and time of day as a random effect. Non-random use of the diel cycle was also tested by comparing the proportions of activity frequency of each period using chi-squared tests.

## Results

A total of 519 banteng images were captured over 36,550 camera trap nights. A total of 497 events were considered to be independent, with 22 discarded due to autocorrelation of individuals and/or multiple captures within the same hour at the same station. A total of 198 events occurred in the wet season whilst 299 events occurred in the dry season. The least captures were obtained in TWR (48 captures/9.3% of the total) however it had the highest survey effort (18,112 nights) and the most camera trap stations (176 stations), whereas the most captures were recorded in MFR (313 captures/63%) (Table 1).

### Diel activity patterns

**Fig 2:**
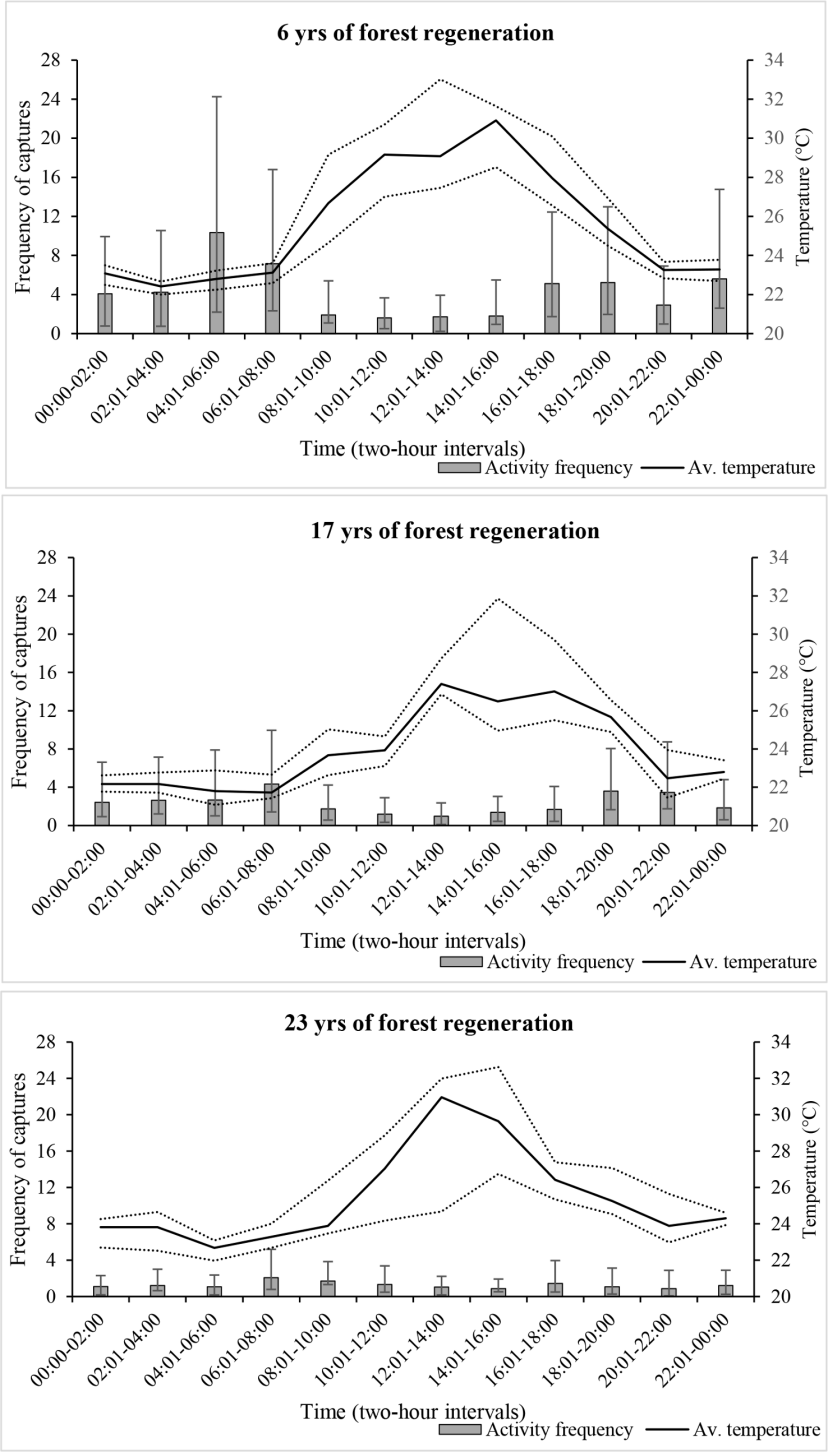
Diel activity patterns of Bornean banteng and forest ambient temperature in Sabah. Activity and corresponding temperature data was collected using camera traps deployed in three forests at different stages of regeneration following logging activity. Activity and temperature data were pooled to two-hour intervals and bootstrapped for 400 iterations to obtain non-parametric 95% confidence intervals around each estimate.

The random intercept GLMM model provided the best fit for the activity data with the lowest AIC values and normalised residual distribution. Correlations between the seasons were low in all forests. Correlations between successive two-hour intervals were low (<0.22) in MFR and MBCABZ, however were high (Hour07-08: 0.53) for hours 12:01–14:00 and 14:01–16:00 in TWR. Refer to Table 2 for GLMM estimates and S1 Table for GLM estimates. There was no evidence to suggest ambient temperature had an effect upon banteng activity in any forest, however, there were indications of a seasonality effect, with less activity during the wet season (z = -2.6, Std. Error = 0.13, p = 0.01) but only in forest with the shortest regeneration time (MFR: 6 years). The GLM bootstrapped estimates of two-hourly activity were plotted according to time of day (Fig 2) and indicated that activity in this same forest was elevated over the dawn and dusk period, however the GLMM estimates (Table 2, Model 1) indicated it was only significantly higher at dawn between hours 04:01–06:00 (Hour03: z = 3.73, Std. Error = 0.24, p = <0.001). During the daylight hours, between 08:01 and 18:00, activity was significantly lower (see Table 2, Model 1: Hour05-Hour08 for estimates). In forest with a longer regeneration time (MBCABZ: 17 years), activity was slightly elevated during the morning (06:01–08:00 hours) and evening (18:01–22:00 hours) although not significantly different. However, during the day, between the hours of 12:01–16:00, activity was considerably lower, particularly during 10:01–12:00 hours (Hour06: z = -2.59, Std. Error = 0.52, p = 0.01) but for a shorter period compared to MFR. Banteng activity was consistent throughout the day in forest with 23 years of regeneration (TWR) but no evidence was found to suggest significant fluctuations occurred at any time, including during the midday hours.

**Table 2:** Model estimates from GLMMs and bootstrapped GLMs of Bornean banteng diel activity patterns analysed alongside ambient temperature, seasonality, time of day and period of the day.

		Malua Forest Reserve							Maliau Basin Conservation Area Buffer Zones						Tabin Wildlife Reserve					
No.	Dependent variable	Parameter	GLMM Estimate	Std. Error	z value	P value	GLM Boot estimate	Boot 95% CI	GLMM Estimate	Std. Error	z value	P value	GLM Boot estimate	Boot 95% CI	GLMM Estimate	Std. Error	z value	P value	GLM Boot estimate	Boot 95% CI
1	Activity frequency	(Intercept)	0.50	1.24	0.41	0.68	-	-	-1.13	1.33	-0.85	0.40	-	-	0.26	2.39	0.11	0.91	-	-
		Temp.	0.05	0.05	0.94	0.35	-	-	0.09	0.06	1.61	0.11	-	-	0.00	0.10	-0.03	0.98	-	-
		SeasonWet	-0.33	0.13	-2.60	**0.01**	-	-	0.03	0.16	0.16	0.88	-	-	-0.36	0.36	-0.98	0.33	-	-
		HourH02	0.08	0.28	0.28	0.78	4.25	8.22–3.5	-0.02	0.35	-0.05	0.96	2.42	2.65–1.43	-	-	-	-	1.22	0.56–8.11
		HourH03	0.91	0.24	3.73	**0.00**	10.33	8.13–19.98	0.11	0.34	0.33	0.74	2.65	2.66–1.64	0.02	0.74	0.03	0.98	1.06	0.88–7.07
		HourH04	0.49	0.26	1.91	0.06	7.17	4.83–13.86	0.44	0.32	1.39	0.16	2.66	4.33–2.90	0.72	0.54	1.32	0.19	2.08	1.29–13.85
		HourH05	-1.05	0.45	-2.33	**0.02**	1.90	0.81–3.67	-0.57	0.43	-1.32	0.19	4.33	1.73–1.16	0.50	0.86	0.58	0.56	1.72	0.40–11.44
		HourH06	-1.36	0.52	-2.59	**0.01**	1.62	3.13–1.62	-0.92	0.53	-1.76	0.08	1.73	1.20–0.86	0.29	0.67	0.44	0.66	1.32	0.84–8.81
		HourH07	-1.33	0.67	-1.97	**0.05**	1.72	1.48–3.12	-1.51	0.71	-2.13	**0.03**	1.20	0.98–0.87	-0.02	0.80	-0.03	0.98	1.04	0.87–6.95
		HourH08	-1.25	0.57	-2.20	**0.03**	1.81	0.86–3.50	-1.36	0.65	-2.08	**0.04**	0.98	1.40–0.95	-0.18	1.34	-0.13	0.90	0.87	0.34–5.77
		HourH09	-0.12	0.42	-0.29	0.77	5.11	3.37–9.88	-0.93	0.54	-1.73	0.08	1.40	1.69–1.25	0.45	0.65	0.69	0.49	1.46	0.96–9.71
		HourH10	0.09	0.32	0.28	0.78	5.23	3.26–10.11	-0.19	0.41	-0.47	0.64	1.69	3.60–1.94	0.21	0.77	0.28	0.78	1.08	0.81–7.23
		HourH11	-0.41	0.32	-1.29	0.20	2.92	1.93–5.64	0.23	0.37	0.64	0.53	3.60	3.46–1.70	-0.20	1.11	-0.18	0.86	0.86	0.78–5.72
		HourH12	0.29	0.28	1.04	0.30	5.61	3.01–10.85	-0.27	0.40	-0.69	0.49	3.46	1.85–1.25	0.19	0.64	0.30	0.77	1.21	0.96–8.04
2	Temperature	(Intercept)	23.00	0.50	45.79	<0.001	-	-	22.17	0.52	42.86	<0.001	-	-	23.40	0.64	36.81	< 0.001	-	-
		HourH02	-0.67	0.67	-0.99	0.32	22.42	22.00–22.66	0.17	0.73	0.23	0.82	22.18	21.72–22.77					23.81	22.52–24.65
		HourH03	-0.33	0.68	-0.49	0.62	22.81	22.25–23.23	0.00	0.73	0.00	1.00	21.80	21.08–22.87	-1.07	1.01	-1.06	0.29	22.68	21.97–23.08
		HourH04	0.17	0.68	0.24	0.81	23.13	22.58–23.60	0.00	0.73	0.00	1.00	21.72	21.44–22.67	0.00	0.90	0.00	1.00	23.29	22.67–24.00
		HourH05	3.75	0.82	4.55	**<0.001**	26.68	24.65–29.17	1.83	0.80	2.29	**<0.05**	23.68	22.64–25.02	0.60	1.59	0.38	0.71	23.89	23.46–26.37
		HourH06	5.80	0.80	7.21	**<0.001**	29.17	27.00–30.70	1.83	0.86	2.13	**<0.05**	23.94	23.11–24.65	3.10	1.03	3.02	**<0.01**	27.04	24.17–28.86
		HourH07	7.50	1.17	6.43	**<0.001**	29.08	27.46–33.02	5.50	1.05	5.24	**<0.001**	27.40	26.86–28.73	4.60	1.06	4.33	**<0.001**	30.96	24.67–31.98
		HourH08	7.50	0.90	8.35	**<0.001**	30.91	28.52–31.65	5.83	0.95	6.12	**<0.001**	26.49	24.96–31.86	8.60	2.05	4.21	**<0.001**	29.64	26.74–32.62
		HourH09	5.40	0.80	6.77	**<0.001**	27.97	26.56–30.10	5.50	0.83	6.65	**<0.001**	27.01	25.50–29.69	3.10	1.03	3.02	**<0.01**	26.42	25.35–27.39
		HourH10	2.80	0.75	3.71	**<0.001**	25.37	24.50–26.94	3.83	0.80	4.81	**<0.001**	25.67	24.89–26.55	2.60	1.29	2.02	**<0.05**	25.25	24.53–27.06
		HourH11	0.33	0.68	0.49	0.63	23.25	22.83–23.67	1.08	0.84	1.29	0.20	22.47	21.46–23.94	0.60	1.59	0.38	0.71	23.89	22.98–25.65
	* *	HourH12	0.40	0.72	0.56	0.58	23.28	22.70–23.78	0.83	0.78	1.06	0.29	22.80	22.45–23.42	0.85	0.97	0.87	0.38	24.30	23.92–24.60
3	Activity frequency	(Intercept)	1.49	0.22	6.68	<0.001	-	-	0.98	0.17	5.85	<0.001	-	-	0.10	0.30	0.32	0.75	-	-
		PeriodCrep.	0.59	0.38	1.56	0.12	-	-	0.13	0.28	0.48	0.63	-	-	0.09	0.51	0.17	0.86	-	-
		PeriodDiur.	-0.36	0.30	-1.18	0.24	-	-	-0.31	0.24	-1.31	0.19	-	-	0.39	0.35	1.12	0.26	-	-

### Diel ambient temperatures

The random intercept model provided the best fit for the ambient temperature data, modelled as a factor of time of day, for each of the three forests (Table 2, Model 2). Correlations between two-hourly temperatures were high between 02:01–08:00 (Hour2-3 and Hour3-4) in both MFR (~0.55) and MBCABZ (0.50). Ambient temperatures were found to fluctuate significantly within each forest during daylight hours; strong significant increases in temperature occurred for an extended period (hours 08:01–20:00) in forest that had the least regeneration time (MFR: 6 years, Fig 2); the highest increase occurred during the hours of 14:01–16:00 (z = 8.35, Std. Error = 0.9, p = <0.001) when temperatures reached a mean 31°C (95% CI = 29–32°C). Raw data, however, indicated that temperatures were capable of reaching 44°C. The morning hours in MBCABZ (17 years regeneration) were cooler compared to MFR, and the significantly hottest period occurred slightly later, at 27°C (95% CI = 25.5–30°C) between16:01–18:00 hours (z = 6.65, Std. Error = 0.83, p = <0.001). Forest with 23 years regeneration (TWR) was even cooler during the morning compared to MFR and MBCABZ, and was hottest, at 29–31°C (95% CI = 27.5–33°C) between midday and the afternoon (12:01–14:00 hours: z = 4.33, Std. Error = 1.03, p = <0.001, and 14:01–16:00 hours,) The period over which significantly higher temperatures occurred in TWR was the shortest of all three regenerating forests.

### Diel activity budgets

Stratification of the activity budgets into three specific behaviours per forest yielded insufficient data to model using GLMMs, therefore only the GLM (S1 Table, models 3–5) bootstrapped two-hourly estimates of each behaviour according to hourly fluctuations, without GLM effect sizes, and their correlations with temperature are discussed.

**Fig 3:**
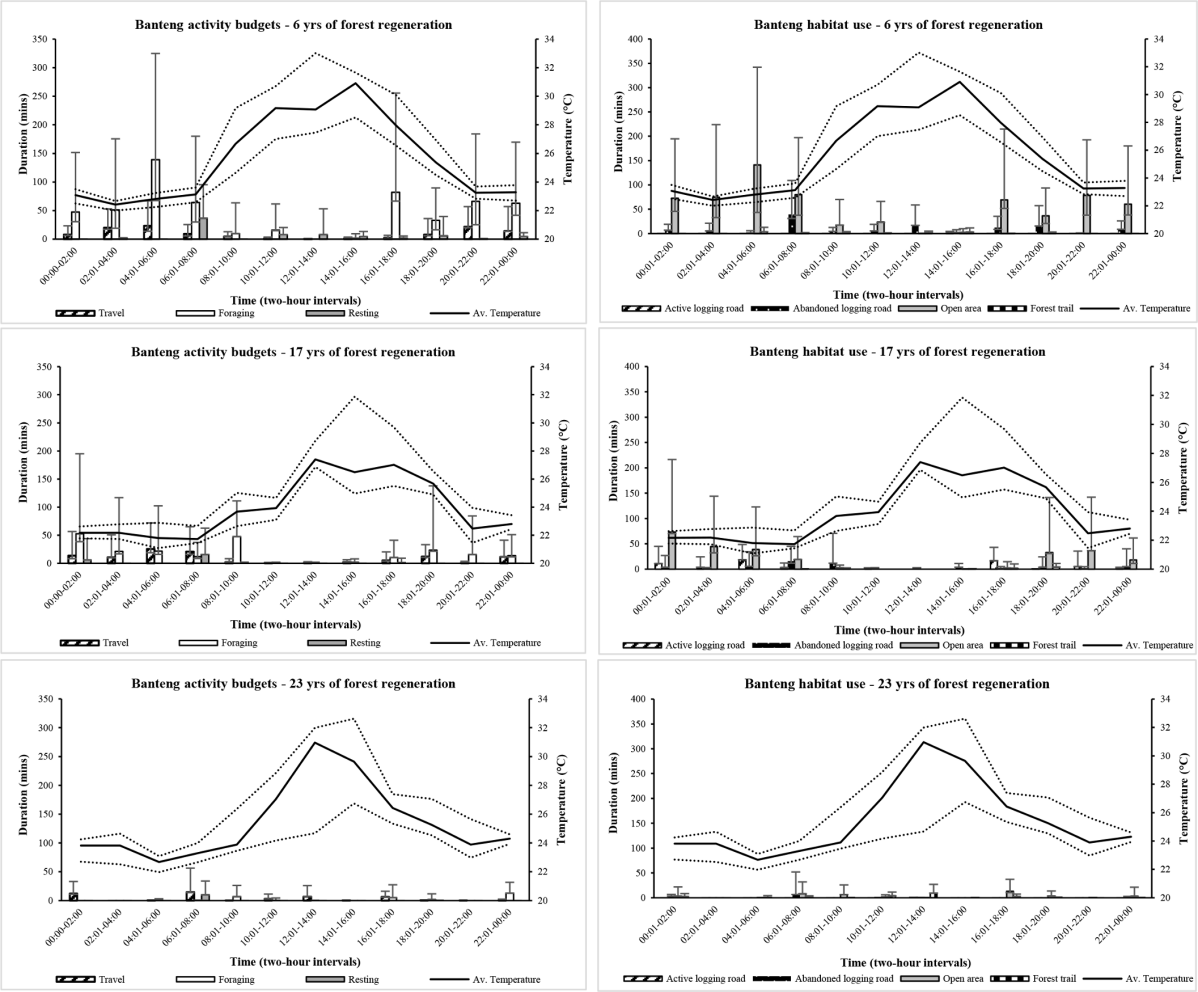
Activity budgets of three specific behaviours, and habitat use of four specific areas, by time and temperature, of Bornean banteng in Sabah recorded using camera traps deployed in three forests, which were at different stages of regeneration following logging activity. Activity, habitat use and temperature data were pooled to two-hour intervals and bootstrapped for 400 iterations to obtain non-parametric 95% confidence intervals around each estimate.

Bantengs spent less time foraging and more time travelling as the forest regenerated from 6 to 23 years, and only a limited time resting (Fig 3). Moving from the shortest to the longest time since logging, the proportion of time spent foraging in a 24-hour period in MFR (6 years) was high (76%) but declined to 62% in MBCABZ (17 years) and again to 34% in TWR (23 years), whilst the reverse was found for the time spent travelling: it increased from 14% in MFR, to 31.5% in MBCABZ, and again to 54% in TWR. There were negative correlations between foraging, travelling and ambient temperature (r_s =_ -0.65, p = <0.05 and r_s_ = -0.69, p = <0.01, respectively) in forest with limited regeneration (MFR: 6 years), and in travelling after 17 years of regeneration (MBCABZ: r_s_ = -0.73, p = <0.01), however after 23 years regeneration (TWR) no behaviours were negatively correlated with ambient temperature. During hours that experienced pronounced heat, there was a tendency for bantengs to rest, however in forest with the longest regeneration time, resting was conducted almost exclusively between 06:01–08:00 hours.

### Diel habitat use

As with the stratified behaviour data, there was insufficient data to model the relationships between habitat use, ambient temperature and time of day using GLMMs. Therefore, we refer only to the GLM (S1 Table, models 6–9) bootstrapped two-hourly estimates for an overview of how the forest is utilised over the course of the day.

Bantengs spent the majority of their time utilising open areas, with limited time spent along old logging roads (Fig 3). In order of increasing regeneration time, bantengs utilised open areas 83% of their time (MFR: 6 years), which declined to 68% (MBCABZ: 17 years), and finally to 53% in TWR after 23 years of regeneration. Conversely, use of forest trails increased from 1.5% and 2%, up to 30% in TWR with the longest regeneration time. Use of open areas was negatively correlated with high ambient temperatures in the two forests with the shortest regeneration times (r_s_ = -0.78, p = <0.01: MFR and r_s_ = -0.63, p = <0.05: MBCABZ). In contrast, use of more shaded forest trails was positively correlated with increasing ambient air temperature in forest with the longest regeneration time (r_s_ = 0.63, p = 0.01). Use of old logging roads was consistent across forests regardless of regeneration time.

### Diel periodic preferences

No relationships were identified in random intercept GLMMs, between activity frequency and period of the day (Table 2, Model 3), to suggest that banteng showed a preference for any one period of the day according to daylight hours. In addition, chi-squared tests did not identify significantly higher proportions in activity for any period.

## Discussion

This study presents the most comprehensive behavioural data set to date for the Bornean banteng. We modelled the spatial and temporal variation of behaviours and habitat use which revealed how they are intricately linked with the diel cycle.

### Behavioural thermoregulation in regenerating logged forests

Bornean bantengs showed adjustment of their activity at specific hours of the diel period, particularly during the midday hours with high temperatures. Whilst we found no evidence to suggest a temperature effect, activity noticeably decreased after sunrise and when ambient temperatures were high, and remained infrequent until sunset. The time spent performing energetic behaviours and utilising exposed areas within the habitat was greatly reduced during that period, and this was most prominent in forest that had the shortest regeneration time and offered the least vegetative cover. Here, banteng maximised energy intake in the morning and evening in open areas, and minimised exposure to high temperatures by seeking refuge in shaded forest. Conversely, in forest with the longest regeneration time, bantengs were active throughout the day but utilised shaded forest trails during the midday period, suggesting the 23 years of regrowth provided some level of thermal respite. Trees are effective in protecting against solar radiation depending on their height, spacing and density [48] but they also provide cooling as moisture evaporates from the leaves [49]. Maintenance of mature forest blocks may conserve the natural cooling environment of humid tropical forests that is essential for tree growth and forest restoration [50].

Banteng utilise open areas for long periods, although usage declines as regeneration time increases. Intensity of use was highest in the 6–17 years of regeneration, and is thought to be due to the prevalence of and preference for pioneer species such as grasses, vines, and shrubs (Ridge, unpublished data), which are outcompeted as the canopy regenerates. We observed a greater investment in foraging behaviour in regenerating forest, and this together with temporary increases in forage and the bantengs’ opportunistic exploitation of degraded areas provides an explanation as to why the body condition of banteng is much higher in forest with short regeneration times [35]. In forest with the longest regeneration time banteng foraged and utilised open areas the least but invested the greatest proportion of time for travelling. A greater search effort may be essential to locate ground-level pioneer vegetation that was probably shaded out as the canopy regenerated and to obtain sufficient forage to meet the energy requirements of their large body size. When forage is limited, ungulates may strip bark [51] for protein and carbohydrates as seen in the sambar deer [52] and Bornean orangutan (*Pongo* spps.) [12]. This behaviour was recorded once in forest that had the longest regeneration time, which almost certainly had fewer internal open areas and least pioneer forage. Additionally, forest edges and roadside verges may offer greater provisions but with a bigger risk of human-wildlife conflict. The exploitation of these areas may indicate a higher tolerance threshold and/or the temporary benefits of increased forage availability outweigh the risk of predation [47]. However, confirming activities in these locations using camera traps is exceptionally difficult due to theft of equipment.

### Adaptive strategies to mitigate heat stress

The strategy of mitigating extreme heat by regulating activity was unfounded for Bornean banteng using the activity frequency pooled according to each forest. The absence of a temperature effect may have been borne out of the geographical differences between the camera trap location and the location of the banteng photographed; cameras were almost exclusively sighted on trees under a, potentially cooler, shaded canopy to avoid unwanted removal, and they overlooked banteng trails that were more exposed along roads and in open areas. For this reason, our extrapolated ambient temperature means are likely to be underestimated, however in the absence of fixed weather stations, the camera traps were invaluable for investigating the animal-climate relationship. The temperature effect may have greater implications for the performance of specific behaviours and habitat use, but insufficient hourly observations prevented a more thorough investigation on this occasion. We did, however, identify preliminary signs that may indicate that Bornean banteng experience thermal stress and exercise behavioural thermoregulation [24] in forests that have been recently logged (0–17 years). Other large-bodied species such as the American bison (*Bison bison*) practice behavioural thermoregulation and adjust daily activity patterns according to changes in temperature and precipitation [25]. For the Asian elephant (*Elephas maximus*), switching activity to the nocturnal period, wallowing or seeking shade are essential to regulate their core body and skin temperature when exposed to the full sun and high temperatures (~31°C) [20]. Temperature highs are detrimental to endotherms; when ambient temperature increases so too does mean body temperature [48], and when in continual locomotion in full sun this can potentially lead to a lethal rise in core body temperatures [20]. Rumination is also primarily limited by ambient temperature [53].

Heat stress in domestic cattle is induced when exposure to temperatures surpass 35°C [54]; observations of raw maximal temperatures in MFR regularly surpassed 35°C, reaching an upper limit of 44°C. Such high temperatures influence metabolic processes and physio-biochemical processes, increasing methane emissions and water intake, and decreasing urination, lactation and body weight, among others [49,54,55]. Black coloured cattle like Bornean banteng bulls are more susceptible to heat, as they absorb more solar radiation and require more frequent use of shade but also exhibit higher rates of heat loss than white and piebald colour cattle [48,56]. Cattle of warm environments have physiological adaptations including increased hair coat thickness and hair weight per unit surface area which are important determinant of non-evaporative heat loss from the body [49]. Slick hair also provides increased thermal resistance due to its association with increased sweating rate and lower metabolic rate [49]. These traits alone are not sufficient to mitigate heat stress though, and even cattle with these adaptations preferentially seek the provision of natural shade by trees [49]. The risk posed by heat stress was probably minor or absent in forest that had regenerated for >2 decades, as the regeneration of the canopy and understorey layer reduced average ambient temperatures and provided better respite against the heat and UV exposure, thus allowing continual activity. Alternative cooling strategies such as bathing or mud wallowing used by the co-existing Sumatran rhinoceros (*Dicerorhinus sumatrensis*) [22] was not observed for the banteng in this study, and this further intensifies the need to conserve dense forest shade and limit the degradation of existing forest habitat.

### Seasonality and diel periodicity of Bornean banteng

We identified a seasonal effect in banteng, whereby activity was reduced during the wet season but only in the forest with the shortest regeneration time. Significantly less activity during the wet season may have been due, in part, to seasonal migration in response to variations in food supply, or flooding of seasonal swamp and lowland riparian forest, displacing bantengs and inducing movement to higher elevations. Seasonality in habitat use, as a result of flooding, is thought to occur in Bornean banteng in TWR [57], and in the Bornean elephant (*E*. *maximus*) along the Kinabatangan River, ~40 km east of our study location in MFR [58]. Flooding in this latter region is severe, and probably caused by extensive loss of natural forest cover and increased non-natural forest cover in the surrounding area [59]; recent logging in MFR may have exacerbated the flooding, and may offer an explanation as to why the seasonality effect was only detected in this location.

We did not detect a preference for any diel period unlike other banteng subspecies in Cambodia (*B*. *j*. *birmanicus*) and Java (*B*. *j*. *javanicus*), and the closely-related gaur (*B*. *gaurus*) in India that were described as being nocturnal [38,47,48]. They also showed contrasting behaviours to coexisting nocturnal sambar deer (*Rusa unicolor*) and the crepuscular or diurnal bearded pig (*Sus barbatus*) which experience the same habitat disturbances [39]. Due to their large body mass, bantengs’ activity may be regulated by their large energy requirements rather than daylight hours. Previous assessment of Javan bantengs suggested activity occurred every two to three hours, alternating between feeding and resting [49].

## Conclusion

In order to achieve more holistic sustainable forest management practices and ensure the provision of ecosystem services and maintenance of biodiversity and ecological processes, as stated in Forest Stewardship Council guidelines, timber management strategies should be broadened to include monitoring of the microclimate because it impacts upon the behaviours of large mammals. Timber harvesting causes extensive habitat perturbation; it causes extensive damage to vegetation and soil compaction along vast tracts of logging roads, increases flood risk and also induces prolonged periods of higher ambient temperatures at ground level, which decline as the forest regenerates. Large mammals enduring logging must also mitigate ensuing habitat and climatic modifications. We observed pronounced responses in banteng activity during the seasons and also during the day, varying the performance of specific behaviours, and shifting habitat use from exposed to shaded habitat when temperatures were high. The incorporation of species behavioural responses and habitat use into timber harvesting practices is important for safeguarding the persistence of large mammals within managed forests, for enhancing ecosystem restoration practices, and for achieving sustainability of both flora and fauna communities. Our results suggest that complete restoration of the forest may not actually be beneficial for large ungulates, and retention of open sites in logged forests that contain pioneer vegetation/suitable forage could actually prove beneficial providing they are secured against subsequent disturbance like hunting activity. Conversely, restoration of the forest reduces water runoff, erosion and flooding, which can be detrimental for habitat quality and availability. Bantengs display signs of adaptability to post-timber harvesting conditions and persist in commercial forests, frequently utilising old logging roads, access roads used by vehicles and degraded open areas for forage and to congregate [34]. Immediate steps taken to reduce stress upon banteng populations in commercial forests could include limiting disturbance along logging roads and open areas during key times of activity, specifically during hours of darkness and the crepuscular period, and reducing timber harvesting activities during the wet season when rainfall is high, flood risk is greater and displacement is more likely. Survival may be facilitated by mindful timber harvesting strategies such as maintenance of a mosaic of mature forest blocks could ensure refuge is continually available for mammals evading habitat disturbances, and for the regulation of core body temperatures and essential physiological functions of endotherms that are ultimately key ecosystem engineers of the forest ecosystem.

## Supporting information

S1 Fig Description of logging methods. Four different methods of logging used in Sabah (Malaysia Borneo) within tropical dipterocarp forests over the past six decades up until present-day: conventional, traditional and newer Reduced Impact Logging (RIL) techniques. Traditional logging methods are more destructive and result in heavy impaction of the substrate, which provides favourable conditions for invasive pioneer species that banteng forage upon.
(TIF)

S1 Table GLM estimates of bootstrapped models. GLMs constructed to obtain two-hourly estimates and 95% confidence intervals for activity patterns of Bornean banteng, ambient temperature, activity budgets (grazing, travelling and other behaviours) and habitat use (Old logging roads, open areas, forest trails and active access roads) in three regenerating forests in Sabah, Malaysia.
(DOCX)

S2 Table Data set. (CSV)
