# Supplementary figures and images for: Spatial and temporal behavioural responses of wild cattle to tropical forest degradation
Source: PLoS One. 2018 Apr 12;13(4):e0195444. doi: 10.1371/journal.pone.0195444 (PMC5896964; doi:10.1371/journal.pone.0195444)

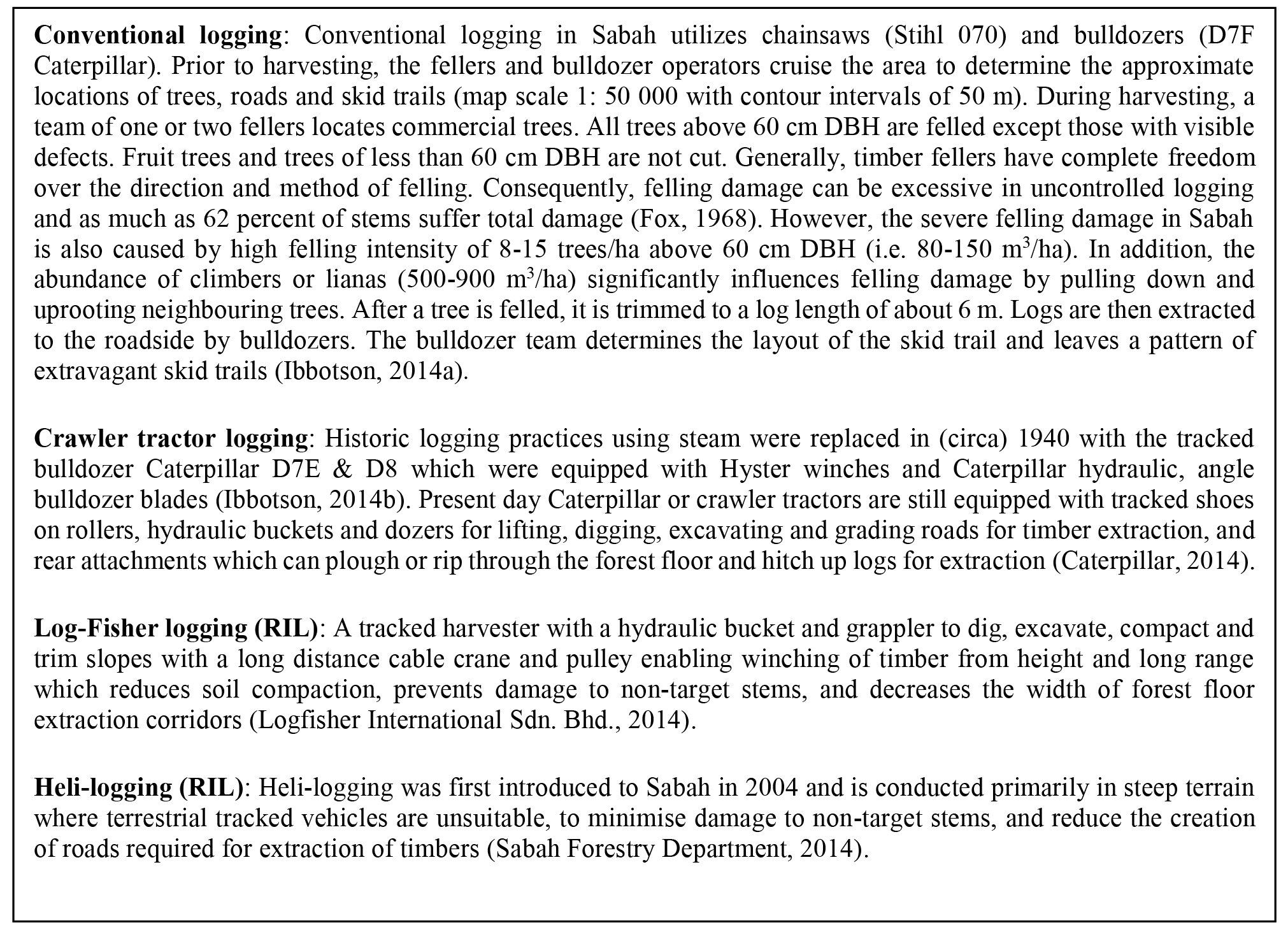

Supplement: S1 Fig — Four different methods of logging used in Sabah (Malaysia Borneo) within tropical dipterocarp forests over the past six decades up until present-day: conventional, traditional and newer Reduced Impact Logging (RIL) techniques. Traditional logging methods are more destructive and result in heavy impaction of the substrate, which provides favourable conditions for invasive pioneer species that banteng forage upon. (TIF) [file pone.0195444.s001.tif]
