# Supplementary material for: Spatial and temporal behavioural responses of wild cattle to tropical forest degradation
Source: PLoS One. 2018 Apr 12;13(4):e0195444. doi: 10.1371/journal.pone.0195444 (PMC5896964; doi:10.1371/journal.pone.0195444)
Supplement: S1 Table — GLMs constructed to obtain two-hourly estimates and 95% confidence intervals for activity patterns of Bornean banteng, ambient temperature, activity budgets (grazing, travelling and other behaviours) and habitat use (Old logging roads, open areas, forest trails and active access roads) in three regenerating forests in Sabah, Malaysia. (DOCX) [file pone.0195444.s002.docx]

S1 Table: **GLM estimates of bootstrapped models**. GLMs constructed to obtain two-hourly estimates and 95% confidence intervals for activity patterns of Bornean banteng, ambient temperature, activity budgets (grazing, travelling and other behaviours) and habitat use (Old logging roads, open areas, forest trails and active access roads) in three regenerating forests in Sabah, Malaysia.

| **Model No.** | **Model description** | **Dependent variable** | **Parameter** | **Estimate** | **Std. Error** | **z value** | **P value** | **Significance** |
| --- | --- | --- | --- | --- | --- | --- | --- | --- |
|  | | | | | | | | |
| 1 | Activity frequency ~ Forest * Time of day + Season | Activity frequency | (Intercept) | 0.95 | 0.27 | 3.54 | 0.00 | *** |
|  |  |  | ForestMFR | 0.64 | 0.33 | 1.95 | 0.05 | . |
|  |  |  | ForestTWR | -0.79 | 0.49 | -1.62 | 0.11 |  |
|  |  |  | HourH02 | 0.13 | 0.37 | 0.37 | 0.72 |  |
|  |  |  | HourH03 | 0.41 | 0.35 | 1.18 | 0.24 |  |
|  |  |  | HourH04 | 0.54 | 0.34 | 1.60 | 0.11 |  |
|  |  |  | HourH05 | -0.24 | 0.40 | -0.60 | 0.55 |  |
|  |  |  | HourH06 | -0.44 | 0.43 | -1.03 | 0.30 |  |
|  |  |  | HourH07 | -1.95 | 0.76 | -2.57 | 0.01 | * |
|  |  |  | HourH08 | -1.54 | 0.64 | -2.42 | 0.02 | * |
|  |  |  | HourH09 | -0.56 | 0.44 | -1.26 | 0.21 |  |
|  |  |  | HourH10 | 0.41 | 0.35 | 1.18 | 0.24 |  |
|  |  |  | HourH11 | -0.24 | 0.40 | -0.60 | 0.55 |  |
|  |  |  | HourH12 | -0.07 | 0.39 | -0.19 | 0.85 |  |
|  |  |  | SeasonWet | -0.36 | 0.11 | -3.13 | 0.00 | ** |
|  |  |  | ForestMFR:HourH02 | -0.17 | 0.45 | -0.37 | 0.71 |  |
|  |  |  | ForestTWR:HourH02 | -17.43 | 1411.36 | -0.01 | 0.99 |  |
|  |  |  | ForestMFR:HourH03 | 0.61 | 0.41 | 1.48 | 0.14 |  |
|  |  |  | ForestTWR:HourH03 | -1.10 | 0.79 | -1.40 | 0.16 |  |
|  |  |  | ForestMFR:HourH04 | 0.04 | 0.41 | 0.10 | 0.92 |  |
|  |  |  | ForestTWR:HourH04 | 0.15 | 0.60 | 0.26 | 0.80 |  |
|  |  |  | ForestMFR:HourH05 | -0.89 | 0.56 | -1.61 | 0.11 |  |
|  |  |  | ForestTWR:HourH05 | -0.16 | 0.76 | -0.22 | 0.83 |  |
|  |  |  | ForestMFR:HourH06 | -0.59 | 0.56 | -1.04 | 0.30 |  |
|  |  |  | ForestTWR:HourH06 | 0.26 | 0.74 | 0.35 | 0.73 |  |
|  |  |  | ForestMFR:HourH07 | 0.41 | 0.88 | 0.46 | 0.64 |  |
|  |  |  | ForestTWR:HourH07 | 0.85 | 1.11 | 0.76 | 0.45 |  |
|  |  |  | ForestMFR:HourH08 | 0.51 | 0.74 | 0.70 | 0.49 |  |
|  |  |  | ForestTWR:HourH08 | -0.25 | 1.25 | -0.20 | 0.84 |  |
|  |  |  | ForestMFR:HourH09 | 0.63 | 0.52 | 1.22 | 0.22 |  |
|  |  |  | ForestTWR:HourH09 | 0.71 | 0.71 | 1.00 | 0.32 |  |
|  |  |  | ForestMFR:HourH10 | -0.24 | 0.43 | -0.56 | 0.58 |  |
|  |  |  | ForestTWR:HourH10 | -0.81 | 0.73 | -1.11 | 0.27 |  |
|  |  |  | ForestMFR:HourH11 | -0.20 | 0.50 | -0.40 | 0.69 |  |
|  |  |  | ForestTWR:HourH11 | -17.06 | 1411.36 | -0.01 | 0.99 |  |
|  |  |  | ForestMFR:HourH12 | 0.43 | 0.46 | 0.94 | 0.35 |  |
|  |  |  | ForestTWR:HourH12 | -0.33 | 0.75 | -0.44 | 0.66 |  |
| **Ambient temperature** | | | | | | | | |
| 2 | Temperature ~ Forest * Time of day + Season | Temperature | (Intercept) | 0.05 | 0.00 | 52.31 | < 2e-16 | *** |
|  |  |  | ForestMFR | 0.00 | 0.00 | -3.41 | 0.00 | *** |
|  |  |  | ForestTWR | 0.00 | 0.00 | -2.49 | 0.01 | * |
|  |  |  | HourH02 | 0.00 | 0.00 | -0.64 | 0.52 |  |
|  |  |  | HourH03 | 0.00 | 0.00 | -2.49 | 0.01 | * |
|  |  |  | HourH04 | 0.00 | 0.00 | -0.64 | 0.52 |  |
|  |  |  | HourH05 | -0.01 | 0.00 | -5.95 | 0.00 | *** |
|  |  |  | HourH06 | -0.01 | 0.00 | -3.76 | 0.00 | *** |
|  |  |  | HourH07 | -0.01 | 0.00 | -6.91 | 0.00 | *** |
|  |  |  | HourH08 | -0.01 | 0.00 | -5.11 | 0.00 | *** |
|  |  |  | HourH09 | -0.01 | 0.00 | -10.80 | < 2e-16 | *** |
|  |  |  | HourH10 | -0.01 | 0.00 | -7.30 | 0.00 | *** |
|  |  |  | HourH11 | 0.00 | 0.00 | -2.97 | 0.00 | ** |
|  |  |  | HourH12 | 0.00 | 0.00 | -1.88 | 0.06 | . |
|  |  |  | SeasonWet | 0.00 | 0.00 | -1.45 | 0.15 |  |
|  |  |  | ForestMFR:HourH02 | 0.00 | 0.00 | 1.30 | 0.20 |  |
|  |  |  | ForestTWR:HourH02 | NA | NA | NA | NA |  |
|  |  |  | ForestMFR:HourH03 | 0.00 | 0.00 | 2.63 | 0.01 | ** |
|  |  |  | ForestTWR:HourH03 | 0.00 | 0.00 | 2.76 | 0.01 | ** |
|  |  |  | ForestMFR:HourH04 | 0.00 | 0.00 | 0.47 | 0.64 |  |
|  |  |  | ForestTWR:HourH04 | 0.00 | 0.00 | 0.00 | 1.00 |  |
|  |  |  | ForestMFR:HourH05 | 0.00 | 0.00 | 1.41 | 0.16 |  |
|  |  |  | ForestTWR:HourH05 | NA | NA | NA | NA |  |
|  |  |  | ForestMFR:HourH06 | 0.00 | 0.00 | -0.83 | 0.41 |  |
|  |  |  | ForestTWR:HourH06 | 0.00 | 0.00 | 1.67 | 0.10 | . |
|  |  |  | ForestMFR:HourH07 | 0.00 | 0.00 | -0.94 | 0.35 |  |
|  |  |  | ForestTWR:HourH07 | 0.00 | 0.00 | 2.01 | 0.05 | * |
|  |  |  | ForestMFR:HourH08 | 0.00 | 0.00 | -1.96 | 0.05 | . |
|  |  |  | ForestTWR:HourH08 | -0.01 | 0.00 | -3.35 | 0.00 | ** |
|  |  |  | ForestMFR:HourH09 | 0.00 | 0.00 | 0.44 | 0.66 |  |
|  |  |  | ForestTWR:HourH09 | 0.01 | 0.00 | 3.73 | 0.00 | *** |
|  |  |  | ForestMFR:HourH10 | 0.00 | 0.00 | 1.00 | 0.32 |  |
|  |  |  | ForestTWR:HourH10 | 0.00 | 0.00 | 0.09 | 0.93 |  |
|  |  |  | ForestMFR:HourH11 | 0.00 | 0.00 | 2.44 | 0.02 | * |
|  |  |  | ForestTWR:HourH11 | NA | NA | NA | NA |  |
|  |  |  | ForestMFR:HourH12 | 0.00 | 0.00 | 1.57 | 0.12 |  |
|  |  |  | ForestTWR:HourH12 | 0.00 | 0.00 | -0.08 | 0.94 |  |
| **Activity budgets** | | | | | | | | |
| 3 | Grazing ~ Forest * Time of day + Season | Grazing | (Intercept) | 3.74 | 0.07 | 56.45 | < 2e-16 | *** |
|  |  |  | ForestMFR | 0.09 | 0.09 | 1.04 | 0.30 |  |
|  |  |  | ForestTWR | -20.02 | 857.65 | -0.02 | 0.98 |  |
|  |  |  | HourH02 | -0.25 | 0.10 | -2.56 | 0.01 | * |
|  |  |  | HourH03 | -0.41 | 0.10 | -4.03 | 0.00 | *** |
|  |  |  | HourH04 | -2.22 | 0.21 | -10.77 | < 2e-16 | *** |
|  |  |  | HourH05 | -0.64 | 0.11 | -5.80 | 0.00 | *** |
|  |  |  | HourH06 | -19.99 | 856.72 | -0.02 | 0.98 |  |
|  |  |  | HourH07 | -19.99 | 856.72 | -0.02 | 0.98 |  |
|  |  |  | HourH08 | -19.99 | 856.72 | -0.02 | 0.98 |  |
|  |  |  | HourH09 | -19.99 | 856.72 | -0.02 | 0.98 |  |
|  |  |  | HourH10 | -0.74 | 0.11 | -6.55 | 0.00 | *** |
|  |  |  | HourH11 | -19.99 | 856.72 | -0.02 | 0.98 |  |
|  |  |  | HourH12 | -2.48 | 0.23 | -10.68 | < 2e-16 | *** |
|  |  |  | SeasonWet | -0.10 | 0.03 | -3.27 | 0.00 | ** |
|  |  |  | ForestMFR:HourH02 | 0.92 | 0.12 | 7.40 | 0.00 | *** |
|  |  |  | ForestTWR:HourH02 | 0.25 | 1212.89 | 0.00 | 1.00 |  |
|  |  |  | ForestMFR:HourH03 | 1.46 | 0.13 | 11.68 | < 2e-16 | *** |
|  |  |  | ForestTWR:HourH03 | 16.31 | 857.65 | 0.02 | 0.98 |  |
|  |  |  | ForestMFR:HourH04 | 2.58 | 0.22 | 11.62 | < 2e-16 | *** |
|  |  |  | ForestTWR:HourH04 | 2.22 | 1212.89 | 0.00 | 1.00 |  |
|  |  |  | ForestMFR:HourH05 | -0.17 | 0.16 | -1.07 | 0.29 |  |
|  |  |  | ForestTWR:HourH05 | 19.50 | 857.65 | 0.02 | 0.98 |  |
|  |  |  | ForestMFR:HourH06 | 19.23 | 856.72 | 0.02 | 0.98 |  |
|  |  |  | ForestTWR:HourH06 | 37.27 | 1212.24 | 0.03 | 0.98 |  |
|  |  |  | ForestMFR:HourH07 | -0.08 | 1211.71 | 0.00 | 1.00 |  |
|  |  |  | ForestTWR:HourH07 | 35.19 | 1212.24 | 0.03 | 0.98 |  |
|  |  |  | ForestMFR:HourH08 | 17.26 | 856.72 | 0.02 | 0.98 |  |
|  |  |  | ForestTWR:HourH08 | 19.99 | 1484.95 | 0.01 | 0.99 |  |
|  |  |  | ForestMFR:HourH09 | 20.33 | 856.72 | 0.02 | 0.98 |  |
|  |  |  | ForestTWR:HourH09 | 38.91 | 1212.24 | 0.03 | 0.97 |  |
|  |  |  | ForestMFR:HourH10 | 0.28 | 0.15 | 1.86 | 0.06 | . |
|  |  |  | ForestTWR:HourH10 | 18.25 | 857.65 | 0.02 | 0.98 |  |
|  |  |  | ForestMFR:HourH11 | 20.56 | 856.72 | 0.02 | 0.98 |  |
|  |  |  | ForestTWR:HourH11 | 19.99 | 1484.95 | 0.01 | 0.99 |  |
|  |  |  | ForestMFR:HourH12 | 2.54 | 0.25 | 10.25 | < 2e-16 | *** |
|  |  |  | ForestTWR:HourH12 | 2.48 | 1212.89 | 0.00 | 1.00 |  |
| 4 | Travelling ~ Forest * Time of day + Season | Travelling | (Intercept) | 0.71 | 0.27 | 2.64 | 0.01 | ** |
|  |  |  | ForestMFR | 2.57 | 0.28 | 9.29 | < 2e-16 | *** |
|  |  |  | ForestTWR | 0.20 | 0.37 | 0.55 | 0.58 |  |
|  |  |  | HourH02 | 2.15 | 0.28 | 7.61 | 0.00 | *** |
|  |  |  | HourH03 | 1.72 | 0.29 | 5.92 | 0.00 | *** |
|  |  |  | HourH04 | 0.13 | 0.37 | 0.37 | 0.72 |  |
|  |  |  | HourH05 | -0.34 | 0.41 | -0.81 | 0.42 |  |
|  |  |  | HourH06 | -0.15 | 0.39 | -0.39 | 0.70 |  |
|  |  |  | HourH07 | -16.14 | 519.22 | -0.03 | 0.98 |  |
|  |  |  | HourH08 | -1.95 | 0.76 | -2.57 | 0.01 | * |
|  |  |  | HourH09 | -0.85 | 0.49 | -1.74 | 0.08 | . |
|  |  |  | HourH10 | 2.76 | 0.28 | 10.03 | < 2e-16 | *** |
|  |  |  | HourH11 | 2.23 | 0.28 | 7.92 | 0.00 | *** |
|  |  |  | HourH12 | 1.52 | 0.30 | 5.15 | 0.00 | *** |
|  |  |  | SeasonWet | 0.37 | 0.05 | 7.46 | 0.00 | *** |
|  |  |  | ForestMFR:HourH02 | -2.22 | 0.30 | -7.38 | 0.00 | *** |
|  |  |  | ForestTWR:HourH02 | -18.43 | 519.78 | -0.04 | 0.97 |  |
|  |  |  | ForestMFR:HourH03 | -2.07 | 0.31 | -6.66 | 0.00 | *** |
|  |  |  | ForestTWR:HourH03 | -4.49 | 1.07 | -4.19 | 0.00 | *** |
|  |  |  | ForestMFR:HourH04 | -0.84 | 0.39 | -2.18 | 0.03 | * |
|  |  |  | ForestTWR:HourH04 | 1.62 | 0.46 | 3.55 | 0.00 | *** |
|  |  |  | ForestMFR:HourH05 | -1.94 | 0.48 | -4.08 | 0.00 | *** |
|  |  |  | ForestTWR:HourH05 | -2.44 | 1.11 | -2.19 | 0.03 | * |
|  |  |  | ForestMFR:HourH06 | -4.43 | 0.81 | -5.45 | 0.00 | *** |
|  |  |  | ForestTWR:HourH06 | 0.94 | 0.50 | 1.89 | 0.06 | . |
|  |  |  | ForestMFR:HourH07 | 11.97 | 519.22 | 0.02 | 0.98 |  |
|  |  |  | ForestTWR:HourH07 | 17.69 | 519.22 | 0.03 | 0.97 |  |
|  |  |  | ForestMFR:HourH08 | -1.25 | 0.84 | -1.49 | 0.14 |  |
|  |  |  | ForestTWR:HourH08 | -0.13 | 1.06 | -0.13 | 0.90 |  |
|  |  |  | ForestMFR:HourH09 | -0.01 | 0.51 | -0.01 | 0.99 |  |
|  |  |  | ForestTWR:HourH09 | 1.17 | 0.59 | 1.98 | 0.05 | * |
|  |  |  | ForestMFR:HourH10 | -4.32 | 0.32 | -13.31 | < 2e-16 | *** |
|  |  |  | ForestTWR:HourH10 | -4.15 | 0.62 | -6.66 | 0.00 | *** |
|  |  |  | ForestMFR:HourH11 | -6.12 | 0.58 | -10.58 | < 2e-16 | *** |
|  |  |  | ForestTWR:HourH11 | -4.31 | 0.80 | -5.38 | 0.00 | *** |
|  |  |  | ForestMFR:HourH12 | -4.71 | 0.47 | -10.11 | < 2e-16 | *** |
|  |  |  | ForestTWR:HourH12 | -2.50 | 0.56 | -4.45 | 0.00 | *** |
| 5 | Other behaviours ~ Forest * Time of day + Season | Other behaviours | (Intercept) | -17.65 | 2782.00 | -0.01 | 1.00 |  |
|  |  |  | ForestMFR | -0.43 | 4009.00 | 0.00 | 1.00 |  |
|  |  |  | ForestTWR | 0.00 | 3935.00 | 0.00 | 1.00 |  |
|  |  |  | HourH02 | 0.00 | 3935.00 | 0.00 | 1.00 |  |
|  |  |  | HourH03 | 0.00 | 3935.00 | 0.00 | 1.00 |  |
|  |  |  | HourH04 | 0.00 | 3935.00 | 0.00 | 1.00 |  |
|  |  |  | HourH05 | 0.00 | 3935.00 | 0.00 | 1.00 |  |
|  |  |  | HourH06 | 0.00 | 3935.00 | 0.00 | 1.00 |  |
|  |  |  | HourH07 | 0.00 | 3935.00 | 0.00 | 1.00 |  |
|  |  |  | HourH08 | 0.00 | 3935.00 | 0.00 | 1.00 |  |
|  |  |  | HourH09 | 20.04 | 2782.00 | 0.01 | 0.99 |  |
|  |  |  | HourH10 | 0.00 | 3935.00 | 0.00 | 1.00 |  |
|  |  |  | HourH11 | 0.00 | 3935.00 | 0.00 | 1.00 |  |
|  |  |  | HourH12 | 0.00 | 3935.00 | 0.00 | 1.00 |  |
|  |  |  | SeasonWet | -3.03 | 0.21 | -14.43 | <2e-16 | *** |
|  |  |  | ForestMFR:HourH02 | 18.88 | 4880.00 | 0.00 | 1.00 |  |
|  |  |  | ForestTWR:HourH02 | 0.00 | 5565.00 | 0.00 | 1.00 |  |
|  |  |  | ForestMFR:HourH03 | 0.00 | 5669.00 | 0.00 | 1.00 |  |
|  |  |  | ForestTWR:HourH03 | 0.00 | 5565.00 | 0.00 | 1.00 |  |
|  |  |  | ForestMFR:HourH04 | 21.86 | 4880.00 | 0.00 | 1.00 |  |
|  |  |  | ForestTWR:HourH04 | 20.73 | 4819.00 | 0.00 | 1.00 |  |
|  |  |  | ForestMFR:HourH05 | 0.00 | 5669.00 | 0.00 | 1.00 |  |
|  |  |  | ForestTWR:HourH05 | 0.00 | 5565.00 | 0.00 | 1.00 |  |
|  |  |  | ForestMFR:HourH06 | 20.19 | 4880.00 | 0.00 | 1.00 |  |
|  |  |  | ForestTWR:HourH06 | 0.00 | 5565.00 | 0.00 | 1.00 |  |
|  |  |  | ForestMFR:HourH07 | 22.13 | 4880.00 | 0.01 | 1.00 |  |
|  |  |  | ForestTWR:HourH07 | 0.00 | 5565.00 | 0.00 | 1.00 |  |
|  |  |  | ForestMFR:HourH08 | 19.42 | 4880.00 | 0.00 | 1.00 |  |
|  |  |  | ForestTWR:HourH08 | 0.00 | 5565.00 | 0.00 | 1.00 |  |
|  |  |  | ForestMFR:HourH09 | -1.16 | 4009.00 | 0.00 | 1.00 |  |
|  |  |  | ForestTWR:HourH09 | -20.04 | 4819.00 | 0.00 | 1.00 |  |
|  |  |  | ForestMFR:HourH10 | 0.00 | 5669.00 | 0.00 | 1.00 |  |
|  |  |  | ForestTWR:HourH10 | 16.86 | 4819.00 | 0.00 | 1.00 |  |
|  |  |  | ForestMFR:HourH11 | 0.00 | 5669.00 | 0.00 | 1.00 |  |
|  |  |  | ForestTWR:HourH11 | 0.00 | 5565.00 | 0.00 | 1.00 |  |
|  |  |  | ForestMFR:HourH12 | 19.57 | 4880.00 | 0.00 | 1.00 |  |
|  |  |  | ForestTWR:HourH12 | 0.00 | 5565.00 | 0.00 | 1.00 |  |
|  |  |  | ForestTWR:HourH12 | 0.00 | 5565.00 | 0.00 | 1.00 |  |
| **Habitat use** | | | | | | | | |
| 6 | Old logging roads ~ Forest * Time of day + Season | Old logging roads | (Intercept) | 3.08 | 0.11 | 26.93 | < 2e-16 | *** |
|  |  |  | ForestMFR | -0.77 | 0.20 | -3.88 | 0.00 | *** |
|  |  |  | ForestTWR | -2.08 | 0.34 | -6.20 | 0.00 | *** |
|  |  |  | HourH02 | -1.90 | 0.31 | -6.13 | 0.00 | *** |
|  |  |  | HourH03 | 0.16 | 0.15 | 1.06 | 0.29 |  |
|  |  |  | HourH04 | -0.39 | 0.18 | -2.23 | 0.03 | * |
|  |  |  | HourH05 | 1.17 | 0.13 | 9.15 | < 2e-16 | *** |
|  |  |  | HourH06 | -2.59 | 0.42 | -6.12 | 0.00 | *** |
|  |  |  | HourH07 | -3.69 | 0.72 | -5.15 | 0.00 | *** |
|  |  |  | HourH08 | -19.82 | 1364.61 | -0.02 | 0.99 |  |
|  |  |  | HourH09 | -2.59 | 0.42 | -6.12 | 0.00 | *** |
|  |  |  | HourH10 | 0.10 | 0.15 | 0.62 | 0.54 |  |
|  |  |  | HourH11 | -1.61 | 0.27 | -5.88 | 0.00 | *** |
|  |  |  | HourH12 | 0.45 | 0.14 | 3.18 | 0.00 | ** |
|  |  |  | SeasonWet | -0.87 | 0.05 | -16.56 | < 2e-16 | *** |
|  |  |  | ForestMFR:HourH02 | 2.11 | 0.38 | 5.56 | 0.00 | *** |
|  |  |  | ForestTWR:HourH02 | -15.84 | 1364.61 | -0.01 | 0.99 |  |
|  |  |  | ForestMFR:HourH03 | -1.83 | 0.44 | -4.16 | 0.00 | *** |
|  |  |  | ForestTWR:HourH03 | -17.90 | 1364.61 | -0.01 | 0.99 |  |
|  |  |  | ForestMFR:HourH04 | 2.30 | 0.25 | 9.23 | < 2e-16 | *** |
|  |  |  | ForestTWR:HourH04 | -17.35 | 1364.61 | -0.01 | 0.99 |  |
|  |  |  | ForestMFR:HourH05 | -1.09 | 0.26 | -4.18 | 0.00 | *** |
|  |  |  | ForestTWR:HourH05 | -18.91 | 1364.61 | -0.01 | 0.99 |  |
|  |  |  | ForestMFR:HourH06 | 2.45 | 0.49 | 5.02 | 0.00 | *** |
|  |  |  | ForestTWR:HourH06 | -15.15 | 1364.61 | -0.01 | 0.99 |  |
|  |  |  | ForestMFR:HourH07 | 4.58 | 0.74 | 6.17 | 0.00 | *** |
|  |  |  | ForestTWR:HourH07 | -14.05 | 1364.61 | -0.01 | 0.99 |  |
|  |  |  | ForestMFR:HourH08 | 18.92 | 1364.61 | 0.01 | 0.99 |  |
|  |  |  | ForestTWR:HourH08 | 2.08 | 1929.85 | 0.00 | 1.00 |  |
|  |  |  | ForestMFR:HourH09 | 3.02 | 0.47 | 6.39 | 0.00 | *** |
|  |  |  | ForestTWR:HourH09 | -15.15 | 1364.61 | -0.01 | 0.99 |  |
|  |  |  | ForestMFR:HourH10 | 0.50 | 0.26 | 1.94 | 0.05 | . |
|  |  |  | ForestTWR:HourH10 | -17.84 | 1364.61 | -0.01 | 0.99 |  |
|  |  |  | ForestMFR:HourH11 | -17.44 | 1364.61 | -0.01 | 0.99 |  |
|  |  |  | ForestTWR:HourH11 | -16.13 | 1364.61 | -0.01 | 0.99 |  |
|  |  |  | ForestMFR:HourH12 | 0.28 | 0.25 | 1.13 | 0.26 |  |
|  |  |  | ForestTWR:HourH12 | -0.97 | 0.54 | -1.80 | 0.07 | . |
| 7 | Open areas ~ Forest * Time of day + Season | Open areas | (Intercept) | 4.32 | 0.05 | 82.71 | < 2e-16 | *** |
|  |  |  | ForestMFR | -0.06 | 0.07 | -0.77 | 0.44 |  |
|  |  |  | ForestTWR | -2.33 | 0.17 | -13.56 | < 2e-16 | *** |
|  |  |  | HourH02 | -0.27 | 0.08 | -3.46 | 0.00 | *** |
|  |  |  | HourH03 | -0.42 | 0.08 | -5.21 | 0.00 | *** |
|  |  |  | HourH04 | -1.46 | 0.12 | -12.38 | < 2e-16 | *** |
|  |  |  | HourH05 | -3.64 | 0.32 | -11.37 | < 2e-16 | *** |
|  |  |  | HourH06 | -19.45 | 518.14 | -0.04 | 0.97 |  |
|  |  |  | HourH07 | -19.45 | 518.14 | -0.04 | 0.97 |  |
|  |  |  | HourH08 | -5.95 | 1.00 | -5.94 | 0.00 | *** |
|  |  |  | HourH09 | -2.73 | 0.21 | -13.21 | < 2e-16 | *** |
|  |  |  | HourH10 | -0.31 | 0.08 | -3.95 | 0.00 | *** |
|  |  |  | HourH11 | -0.30 | 0.08 | -3.87 | 0.00 | *** |
|  |  |  | HourH12 | -0.97 | 0.10 | -9.93 | < 2e-16 | *** |
|  |  |  | SeasonWet | -0.37 | 0.03 | -14.26 | < 2e-16 | *** |
|  |  |  | ForestMFR:HourH02 | 0.46 | 0.11 | 4.41 | 0.00 | *** |
|  |  |  | ForestTWR:HourH02 | -16.84 | 518.14 | -0.03 | 0.97 |  |
|  |  |  | ForestMFR:HourH03 | 1.23 | 0.10 | 11.96 | < 2e-16 | *** |
|  |  |  | ForestTWR:HourH03 | -3.19 | 1.02 | -3.14 | 0.00 | ** |
|  |  |  | ForestMFR:HourH04 | 1.81 | 0.14 | 13.28 | < 2e-16 | *** |
|  |  |  | ForestTWR:HourH04 | 1.74 | 0.25 | 7.02 | 0.00 | *** |
|  |  |  | ForestMFR:HourH05 | 2.41 | 0.34 | 7.10 | 0.00 | *** |
|  |  |  | ForestTWR:HourH05 | 3.70 | 0.39 | 9.38 | < 2e-16 | *** |
|  |  |  | ForestMFR:HourH06 | 18.30 | 518.14 | 0.04 | 0.97 |  |
|  |  |  | ForestTWR:HourH06 | 2.33 | 732.76 | 0.00 | 1.00 |  |
|  |  |  | ForestMFR:HourH07 | 14.25 | 518.14 | 0.03 | 0.98 |  |
|  |  |  | ForestTWR:HourH07 | 2.33 | 732.76 | 0.00 | 1.00 |  |
|  |  |  | ForestMFR:HourH08 | 2.76 | 1.04 | 2.67 | 0.01 | ** |
|  |  |  | ForestTWR:HourH08 | -11.17 | 518.14 | -0.02 | 0.98 |  |
|  |  |  | ForestMFR:HourH09 | 3.17 | 0.22 | 14.62 | < 2e-16 | *** |
|  |  |  | ForestTWR:HourH09 | 3.62 | 0.28 | 12.72 | < 2e-16 | *** |
|  |  |  | ForestMFR:HourH10 | -0.12 | 0.12 | -1.07 | 0.29 |  |
|  |  |  | ForestTWR:HourH10 | 0.41 | 0.24 | 1.72 | 0.09 | . |
|  |  |  | ForestMFR:HourH11 | 0.45 | 0.11 | 4.28 | 0.00 | *** |
|  |  |  | ForestTWR:HourH11 | -16.81 | 518.14 | -0.03 | 0.97 |  |
|  |  |  | ForestMFR:HourH12 | 1.11 | 0.12 | 9.12 | < 2e-16 | *** |
|  |  |  | ForestTWR:HourH12 | 0.94 | 0.25 | 3.71 | 0.00 | *** |
| 8 | Forest trails ~ Forest * Time of day + Season | Forest trails | (Intercept) | -18.05 | 2236.00 | -0.01 | 0.99 |  |
|  |  |  | ForestMFR | 0.27 | 3108.00 | 0.00 | 1.00 |  |
|  |  |  | ForestTWR | 19.06 | 2236.00 | 0.01 | 0.99 |  |
|  |  |  | HourH02 | 0.00 | 3162.00 | 0.00 | 1.00 |  |
|  |  |  | HourH03 | 0.00 | 3162.00 | 0.00 | 1.00 |  |
|  |  |  | HourH04 | 17.12 | 2236.00 | 0.01 | 0.99 |  |
|  |  |  | HourH05 | 17.81 | 2236.00 | 0.01 | 0.99 |  |
|  |  |  | HourH06 | 0.00 | 3162.00 | 0.00 | 1.00 |  |
|  |  |  | HourH07 | 0.00 | 3162.00 | 0.00 | 1.00 |  |
|  |  |  | HourH08 | 16.42 | 2236.00 | 0.01 | 0.99 |  |
|  |  |  | HourH09 | 18.62 | 2236.00 | 0.01 | 0.99 |  |
|  |  |  | HourH10 | 19.86 | 2236.00 | 0.01 | 0.99 |  |
|  |  |  | HourH11 | 0.00 | 3162.00 | 0.00 | 1.00 |  |
|  |  |  | HourH12 | 0.00 | 3162.00 | 0.00 | 1.00 |  |
|  |  |  | SeasonWet | -2.47 | 0.36 | -6.87 | 0.00 | *** |
|  |  |  | ForestMFR:HourH02 | 0.00 | 4395.00 | 0.00 | 1.00 |  |
|  |  |  | ForestTWR:HourH02 | -19.06 | 3872.00 | -0.01 | 1.00 |  |
|  |  |  | ForestMFR:HourH03 | 20.14 | 3828.00 | 0.01 | 1.00 |  |
|  |  |  | ForestTWR:HourH03 | -2.64 | 3162.00 | 0.00 | 1.00 |  |
|  |  |  | ForestMFR:HourH04 | 1.03 | 3108.00 | 0.00 | 1.00 |  |
|  |  |  | ForestTWR:HourH04 | -17.81 | 2236.00 | -0.01 | 0.99 |  |
|  |  |  | ForestMFR:HourH05 | 1.18 | 3108.00 | 0.00 | 1.00 |  |
|  |  |  | ForestTWR:HourH05 | -20.45 | 2236.00 | -0.01 | 0.99 |  |
|  |  |  | ForestMFR:HourH06 | 18.15 | 3828.00 | 0.01 | 1.00 |  |
|  |  |  | ForestTWR:HourH06 | 0.31 | 3162.00 | 0.00 | 1.00 |  |
|  |  |  | ForestMFR:HourH07 | 19.35 | 3828.00 | 0.01 | 1.00 |  |
|  |  |  | ForestTWR:HourH07 | 0.97 | 3162.00 | 0.00 | 1.00 |  |
|  |  |  | ForestMFR:HourH08 | 3.51 | 3108.00 | 0.00 | 1.00 |  |
|  |  |  | ForestTWR:HourH08 | -19.06 | 2236.00 | -0.01 | 0.99 |  |
|  |  |  | ForestMFR:HourH09 | -1.57 | 3108.00 | 0.00 | 1.00 |  |
|  |  |  | ForestTWR:HourH09 | -18.78 | 2236.00 | -0.01 | 0.99 |  |
|  |  |  | ForestMFR:HourH10 | -1.20 | 3108.00 | 0.00 | 1.00 |  |
|  |  |  | ForestTWR:HourH10 | -21.80 | 2236.00 | -0.01 | 0.99 |  |
|  |  |  | ForestMFR:HourH11 | 0.00 | 4395.00 | 0.00 | 1.00 |  |
|  |  |  | ForestTWR:HourH11 | -19.06 | 3872.00 | -0.01 | 1.00 |  |
|  |  |  | ForestMFR:HourH12 | 0.00 | 4395.00 | 0.00 | 1.00 |  |
|  |  |  | ForestTWR:HourH12 | -1.25 | 3162.00 | 0.00 | 1.00 |  |
| 9 | Active access roads ~ Forest * Time of day + Season | Active access roads | (Intercept) | 2.88 | 0.11 | 26.05 | < 2e-16 | *** |
|  |  |  | ForestMFR | -21.48 | 2325.15 | -0.01 | 0.99 |  |
|  |  |  | ForestTWR | -21.48 | 2325.15 | -0.01 | 0.99 |  |
|  |  |  | HourH02 | -21.41 | 2323.64 | -0.01 | 0.99 |  |
|  |  |  | HourH03 | -0.84 | 0.16 | -5.36 | 0.00 | *** |
|  |  |  | HourH04 | -1.22 | 0.18 | -6.80 | 0.00 | *** |
|  |  |  | HourH05 | -21.41 | 2323.64 | -0.01 | 0.99 |  |
|  |  |  | HourH06 | -2.83 | 0.36 | -7.79 | 0.00 | *** |
|  |  |  | HourH07 | -21.41 | 2323.64 | -0.01 | 0.99 |  |
|  |  |  | HourH08 | -21.41 | 2323.64 | -0.01 | 0.99 |  |
|  |  |  | HourH09 | -0.94 | 0.16 | -5.82 | 0.00 | *** |
|  |  |  | HourH10 | -4.91 | 1.00 | -4.90 | 0.00 | *** |
|  |  |  | HourH11 | -21.41 | 2323.64 | -0.01 | 0.99 |  |
|  |  |  | HourH12 | -2.43 | 0.30 | -8.06 | 0.00 | *** |
|  |  |  | SeasonWet | 0.43 | 0.12 | 3.69 | 0.00 | *** |
|  |  |  | ForestMFR:HourH02 | 21.41 | 4026.40 | 0.01 | 1.00 |  |
|  |  |  | ForestTWR:HourH02 | 21.41 | 4026.40 | 0.01 | 1.00 |  |
|  |  |  | ForestMFR:HourH03 | 0.84 | 3288.25 | 0.00 | 1.00 |  |
|  |  |  | ForestTWR:HourH03 | 0.84 | 3288.25 | 0.00 | 1.00 |  |
|  |  |  | ForestMFR:HourH04 | 1.22 | 3288.25 | 0.00 | 1.00 |  |
|  |  |  | ForestTWR:HourH04 | 1.22 | 3288.25 | 0.00 | 1.00 |  |
|  |  |  | ForestMFR:HourH05 | 21.41 | 4026.40 | 0.01 | 1.00 |  |
|  |  |  | ForestTWR:HourH05 | 21.41 | 4026.40 | 0.01 | 1.00 |  |
|  |  |  | ForestMFR:HourH06 | 2.83 | 3288.25 | 0.00 | 1.00 |  |
|  |  |  | ForestTWR:HourH06 | 2.83 | 3288.25 | 0.00 | 1.00 |  |
|  |  |  | ForestMFR:HourH07 | 21.41 | 4026.40 | 0.01 | 1.00 |  |
|  |  |  | ForestTWR:HourH07 | 39.30 | 3287.18 | 0.01 | 0.99 |  |
|  |  |  | ForestMFR:HourH08 | 21.41 | 4026.40 | 0.01 | 1.00 |  |
|  |  |  | ForestTWR:HourH08 | 21.41 | 4026.40 | 0.01 | 1.00 |  |
|  |  |  | ForestMFR:HourH09 | 0.94 | 3288.25 | 0.00 | 1.00 |  |
|  |  |  | ForestTWR:HourH09 | 0.94 | 3288.25 | 0.00 | 1.00 |  |
|  |  |  | ForestMFR:HourH10 | 4.91 | 3288.25 | 0.00 | 1.00 |  |
|  |  |  | ForestTWR:HourH10 | 4.91 | 3288.25 | 0.00 | 1.00 |  |
|  |  |  | ForestMFR:HourH11 | 21.41 | 4026.40 | 0.01 | 1.00 |  |
|  |  |  | ForestTWR:HourH11 | 21.41 | 4026.40 | 0.01 | 1.00 |  |
|  |  |  | ForestMFR:HourH12 | 2.43 | 3288.25 | 0.00 | 1.00 |  |
|  |  |  | ForestTWR:HourH12 | 2.43 | 3288.25 | 0.00 | 1.00 |  |
